# Carbon Nanotubes Interference with Luminescence-Based Assays

**DOI:** 10.3390/ma13194270

**Published:** 2020-09-25

**Authors:** Tomasz Szymański, Marcelina Kempa, Michael Giersig, Jakub Dalibor Rybka

**Affiliations:** 1 Center for Advanced Technology, Adam Mickiewicz University, Uniwersytetu Poznańskiego 10 Street, 61-614 Poznan, Poland; tszymanski@amu.edu.pl (T.S.); marcelina.kempa@op.pl (M.K.); giersig@amu.edu.pl (M.G.); 2 Faculty of Chemistry, Adam Mickiewicz University, Uniwersytetu Poznańskiego 8 Street, 61-614 Poznan, Poland; 3 Faculty of Biology, Adam Mickiewicz University, Uniwersytetu Poznańskiego 6, 61-614 Poznań, Poland; 4 Department of Physics, Institute of Experimental Physics, Freie Universität, Arnimallee 14, 14195 Berlin, Germany

**Keywords:** tissue engineering, biomaterials, carbon nanotubes, scaffolds, cytotoxicity, chondrocytes

## Abstract

Carbon nanotubes (CNTs) are one of the most promising nanomaterials synthesized to date. Thanks to their unique mechanical, electronic, and optical properties, they have found a wide application in electronics in the production of biosensors and nanocomposites. The functionalization of multiwalled carbon nanotubes (MWCNTs) is aimed at making them biocompatible by adding hydrophilic groups on their surface, increasing their solubility and thus rendering them applicable in the regenerative medicine. So far, there is conflicting information about carbon nanotubes in biological systems. This paper investigates the effect of functionalized, oxidized, multiwalled carbon nanotubes (MWCNT-Ox) on the cytotoxicity of normal human articular chondrocytes (NHAC-kn cell line). Since absorbance-based and fluorescence-based assays were shown to interfere with carbon nanotubes, luminescence-based tests were carried out, as they work on a different method of detection and provide advantages over the mentioned ones. Cell viability and reactive oxygen species (ROS) tests were carried out. The cell viability assay showed that with the increasing MWCNTs concentration, the number of viable chondrocytes was significantly decreasing. Exposure to MWCNT-Ox indicated oxidative stress in the lowest investigated concentration with a decreased amount of ROS with higher concentrations. However, control experiments with adenosine triphosphate (ATP) and H_2_O_2_—molecules that are detected by the assays—showed that carbon nanotubes interfere directly with measurement, thus rendering the results unreliable. To understand the exact interference mechanisms, further studies must be taken. In conclusion, this study shows that luminescence-based tests yield erroneous results, confirming that in vitro experiments in the literature concerning carbon nanotubes should be analyzed with caution.

## 1. Introduction

### 1.1. Carbon Nanotubes (CNTs)

Carbon nanotubes (CNTs) are allotropic forms of carbon, made of cylindrical folded graphene layers discovered by Sumio Iijima in 1991 [1]. They constitute a diverse group of nanomaterials in terms of structure, size, shape, and properties. The carbon nanotube has a honeycomb-shaped hexagonal graphene network that periodically repeats in space, which gives CNTs impressive mechanical, physico-chemical, and optical properties. The diameter of nanotubes is expressed in nanometers, while the length of nanotubes can be up to several millimeters. The ratio of their length to the diameter is in the range of 10:3 to 10:5 [2,3,4]. There are two types of carbon nanotubes: single-walled carbon nanotubes (SWCNTs) and multiwalled carbon nanotubes (MWCNTs). SWCNTs are made of a single, cylindrical rolled graphene layer. The diameter of most single-walled carbon nanotubes reaches 1 nm. MWCNTs are made of many concentrically rolled individual graphene layers forming a cylindrical structure [5].

Due to the unique properties, nanotubes have enormous application potential and are the subject of research for scientists around the world. The mechanical properties of CNTs result from the combination of rigidity, strength, and flexibility; they also possess exceptional conductive and thermal properties [5,6]. Therefore, they are applied in many fields, such as materials science [7], medicine [8,9,10,11] electronics [12], and energy storage [13]. 

### 1.2. CNTs Toxicity

Since nanotechnology is one of the dynamically developing fields, and its products can be widely used in medicine, the food industry, and electronics, it is necessary to know exactly the nature and impact of CNTs on the health and functioning of the body. This is especially important for the biomedical use of carbon nanotubes. In recent years, researchers’ interest in the use of scaffolds from CNTs for the production of implants has grown, among others, for the treatment of cartilage injuries [14,15,16,17,18]. Carbon nanotubes are attractive for this type of application due to their very high mechanical strength, excellent flexibility, and size, similar to extracellular matrix (ECM) molecules [19]. There is a number of studies that show the positive impact of CNTs on cells, stimulating their proliferation and proper metabolism [20,21,22,23]. However, it was noticed that 3D cultured chondrocytes showed higher tolerance to increasing concentration of carbon nanotubes, compared to 2D culture in which viability decreased with increasing amounts of CNTs. Differences in cell survival in 2D cultures may be caused by carbon nanotubes diffusion into the medium, which facilitates cellular uptake by endocytosis. In 3D culture, carbon nanotubes are embedded in a hydrogel matrix, which limits their ability to be absorbed by the cells [24]. On the other hand, there is much research, carried out either in vitro or in vivo showing that carbon nanotubes are toxic to cells in many aspects [25,26,27].

It was shown that size [28], level of aggregation [29], dose [30], surface modification [31], and purity [32] all have an impact on toxicity. It was also shown that CNTs may cause reactive oxygen species (ROS) production [33]. Carbon nanotubes are characterized by a highly hydrophobic character and very poor solubility in polar and non-polar solvents. Additionally, after synthesis, carbon nanotubes are contaminated with large amounts of amorphous carbon and catalyst particles, e.g., iron, nickel [34].

The initial methods of purification and functionalization of nanotubes are designed to introduce changes in their structure and increase solubility, e.g., in aqueous solutions, which makes it possible to use CNTs in biological systems [19].

### 1.3. CNTs Interactions with Biological Assays

When determining the influence of CNTs on a cell in vitro, the well-established assays are often performed, such as MTT, MTS, WST-1, and LDH measurement. As was described, CNTs interact with a plethora of molecules. The nature of these interactions has been researched and is attributed mainly to van der Waals forces and π–π stacking [35]. Some studies that show interference with widely used molecular assays based on absorbance and fluorescence. Wörle-Knirsch et al. performed MTT assay on A549 cells with various doses of SWCNTs and a strong cytotoxic effect was observed of almost 60% [36]. However, different tests—WST-1, LDH assay, mitochondrial membrane potential, and Annexin V measured by flow cytometry—have not confirmed these results and showed that SWCNTs have a negligible effect on cellular function. Interestingly, MTT and WST-1 tests are based on the same principle—they measure the absorption of the formazan crystals formed by the reduction of tetrazolium substrate by living cells. The only difference between these two tests is that in MTT assay, the end product is water-insoluble, contrary to the WST-1 test, where formed crystals are hydrophilic. Researchers found in TEM images precipitates attached to the SWCNTs that were treated with the MTT test, and no such precipitates on SWCNTs from the WST-1 test. They attribute it to the strong adsorption of insoluble crystals to the surface of carbon nanotubes, yielding false-positive results, since adsorbed crystals are not detected during measurement. Casey et al. assessed the interaction of SWCNTs with a broad panel of indicator dyes that are used routinely for the cellular viability measurement—Comassie Blue, Alamar Blue^TM^ (AB), Neutral Red (NR), MTT, and WST-1—on the same A549 cell line [37]. He determined by spectroscopic measurements that SWCNTs interact with varying degrees with each of those tests, even WST-1 assay, resulting potentially in false-positive outcomes. Such results cast doubt on the validity of such experiments carried out in other research.

The investigated tests are based either on absorbance or fluorescence measurement. These are well-established, relatively cheap, widely used assays that are suitable for use in multiwell-plate formats and high-throughput screening. Since carbon nanotubes are heavily researched in the biomedical and bioengineering field and consensus about their toxicity is not yet reached, there is a need to have such a tool that could be applied for carbon nanotubes experiments and would not interfere with the tested compound. In terms of existing technologies, there is one more, which to our knowledge was not tested with carbon nanotubes: luminescence-based assays. Here, the luminescent signal is generated upon the interaction of recombinant luciferase with the measured substance. There are a few advantages of this technology compared to absorbance and fluorescence-based assays: (1) luminescence is a continuous light source, which is produced ‘in situ’, without the need for an external light source, (2) the signal generated by existing recombinant luciferases are stable for hours, (3) very high sensitivity, because the emitted light is broad across many wavelengths (with a peak emission wavelength λ = 565 nm), and since eukaryotic cells do not intrinsically produce light, sample background is very low [38].

Taking into account these features, such assays seems promising to use with carbon nanotubes.

The purpose of this work is to measure the impact of functionalized, multiwalled carbon nanotubes (MWCNT-Ox) on the viability and reactive oxygen species using luminescent-based tests and determine whether MWCNTs interact with the luminescent signal generated in the assays.

## 2. Materials and Methods

### 2.1. MWCNTs

MWCNTs with a diameter of 15–30 nm, length of 15–20 μm, and purity of up to 95% produced by chemical vapor deposition (CVD) were supplied by Nanolab Inc., Boston, MA, USA

### 2.2. MWCNTs Functionalization

MWCNTs were functionalized to oxidize their surface according to the following method. First, 30 mg of MWCNTs were sonicated at 70 °C in a mixture of concentrated sulfur VI (H_2_SO_4_) and nitric V (HNO_3_) acids in a 3:1 ratio. Then, the mixture was neutralized with 300 mL of 3M sodium hydroxide (NaOH). The purification of oxidized carbon nanotubes was carried out through a centrifugation cycle at the speed 9000× *g*, 20 °C for (a) 15 min, (b) 30 min, and (c) 40 min, with subsequent centrifugation at 12,000× *g*, 4 °C for 40 min. The resulting carbon nanotubes solution was dried using a vacuum evaporator. MWCNTs were suspended in phosphate buffer (PBS). The thermogravimetric method determined the mass of nanotubes in a given volume of solution, and on this basis, the MWCNT-Ox concentration was determined.

### 2.3. MWCNTs Characterization

MWCNTs were characterized using scanning electron microscopy (SEM) to confirm their size and subsequent energy-dispersive X-ray spectroscopy (EDS) analysis to check their purity before and after functionalization. The experiments were carried out on high-resolution Quanta 250 FEG, FEI, microscope. Diameters of MWCNTs were measured with ImageJ software. Fourier-transform infrared spectroscopy (FTIR,) was performed to check the level of functionalization. Briefly, 2 mg of dried MWCNTs and functionalized, oxidized, multiwalled carbon nanotubes (MWCNTs-Ox) were mixed with 250 mg of KBr. The mixture was pressed to form a pellet and was degassed under vacuum. The pellet was analyzed on a Jasco 4700A spectrometer (Jasco Corporation, Tokio, Japan), and the IR spectra were obtained within the range of 4000–400 cm^−1^ with the resolution of 1 cm^−1^.

### 2.4. Cell Culture

The studies were carried out on the established NHAC-kn normal human articular cartilage cell line (Lonza Group, Basel, Switzerland). Cells were cultured in full culture medium composed of DMEM (Corning Inc., Corning, NY, USA) supplemented with 10% fetal bovine serum (FBS) (Corning), 1% penicillin/streptomycin (Corning), and 50 µg/mL 2-phospho-L-ascorbic acid trisodium salt (Sigma Aldrich), at 37 °C and 5% CO_2_. Cells were grown on 75 cm^2^ flasks, and when confluence reached ≈80%, cells were detached with TrypLE™ Express Enzyme (Thermo Fisher Scientific, Waltham, MA, USA. For experiments, cells were plated at the density of 1 × 10^4^ cells/well in opaque, white 96-well plates with the clear bottom in a volume of full culture medium specified in the manufacturers’ protocol. Cells were allowed to attach and grow for 24 h. After this time, growth medium was discarded and replaced with fresh medium or solutions of appropriate concentrations (0.0625 mg/mL, 0.25 mg/mL, 0.5 mg/mL, 1 mg/mL), diluted in full fresh medium.

### 2.5. Viability Test

The test used in the experiment was CellTiter-Glo^®^ Luminescent Cell Viability Assay (Promega, Madison, WI, USA). It determines the number of viable, metabolically active cells based on the quantitative ATP assay. As a result of cell lysis, ATP is released and a luminescent signal is generated, which is proportional to the number of live cells.

The experiment was carried out according to the manufacturer protocol. NHAC-kn cells were plated on an opaque, white 96-well plate with clear bottom (1 × 10^4^ cells/well) with MWCNTs-Ox concentrations: 0.0625 mg/mL, 0.25 mg/mL, 0.5 mg/mL, and 1 mg/mL in a volume of 100 µL/well and incubated for 48 h at 37 °C and 5% CO_2_. Doxorubicin at a concentration of 350 μg/mL was used as a positive control [39]. After 48 h incubation, the assay was performed. The luminescent signal was read (from the top) using a microplate reader (Infinite^®^ 200 PRO, TECAN, Männedorf, Switzerland).

### 2.6. ROS Assay

To determine the reactive oxygen species production, ROS-Glo™ H_2_O_2_ Assay (Promega, Madison, WI, USA) was used. The test is based on a substrate that reacts directly with hydrogen peroxide to form the luciferin precursor. The addition of recombinant luciferase and D-cysteine leads to the conversion of the luciferin precursor into luciferin, which then reacts with the enzyme to generate a luminescent signal. The luminescence signal is proportional to the amount of H_2_O_2_. The experiment was carried out according to the manufacturer’s protocol. NHAC-kn cells were plated on an opaque, white 96-well plate with a clear bottom (1 × 10^4^ cells/well) with MWCNTs-Ox concentrations of 0.0625, 0.25, 0.5, and 1 mg/mL in a volume of 80 µL/well and incubated 24 h at 37 °C and 5% CO_2_. Menadione at a concentration of 100 μM was used as a positive control and added 8 h before measurement. After 48 h, the level of ROS was measured, using a microplate reader (Infinite^®^ 200 PRO, TECAN, Männedorf, Switzerland).

### 2.7. Measurement of MWCNTs Absorption and Luminescence Spectra

To determine the absorption spectra of MWCNT-Ox, 100 µL of 0.0625, 0.25, 0.5, and 1 mg/mL MWCNT-Ox solutions diluted in growth medium were put into a 96-well plate, and the absorbance was measured within the range of 260 to 1000 nm, with 2 nm intervals. As a reference, PBS and growth medium in the same amount as in the sample were used. The measured optical densities (ODs) were subtracted from the MWCNT-Ox ODs. Measurements were performed on an Infinite^®^ 200 PRO, TECAN (Männedorf, Switzerland) microplate reader.

Luminescence spectra were obtained on a Cary Eclipse Spectrofluorimeter (Agilent, Santa Clara, CA, USA) for the same concentrations of MWCNTs-Ox, diluted in PBS. First, 30 µL of 1 µM ATP solution and 100 µL of CellTiter-Glo^®^ reagent was added to MWCNTs solutions. After 10 min, 100 µL of the prepared solutions were added to a quartz cuvette, and the measurement of emitted light was performed, with the emission range from 400 to 700 nm, 1 nm interval. Intensities of light were expressed in arbitrary units (A.U.).

### 2.8. MWCNTs Interference with Luminescence

For CellTiter-Glo^®^ Luminescent Cell Viability Assay, 100 μL of MWCNTs-Ox in concentrations of 0.0625, 0.25, 0.5, and 1 mg/mL was incubated with 1 μM ATP solution for 24 h at 37 °C and 5% CO_2_. The ATP concentration was taken from the manufacturers’ protocol, as it is suggested to be used for a standard curve preparation. Accordingly, for the interference with ROS-Glo ™ H_2_O_2_ Assay, 10 μM of H_2_O_2_ was added to the MWCNTs solutions. The test was performed immediately after the addition of H_2_O_2_ due to its chemical instability. The luminescent signal was read using the Infinite^®^ 200 PRO, TECAN microplate reader.

## 3. Results

### 3.1. MWCNTs Characterization

**Figure 1:**
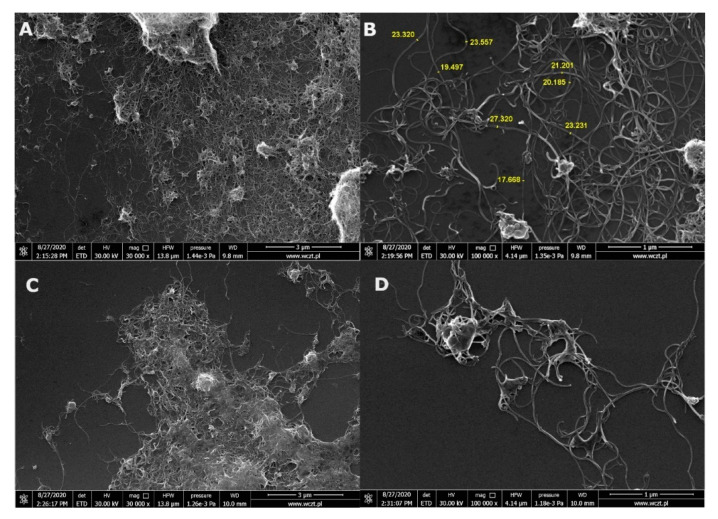
Scanning electron microscopy (SEM) images of both multiwalled carbon nanotubes (MWCNTs) (**A**,**B**) and functionalized oxidized, multiwalled carbon nanotubes (MWCNT-Ox) (**C**,**D**).

SEM of both MWCNTs (Figure 1A,B) and functionalized MWCNT-Ox (Figure 1C,D), confirmed the diameters of the CNTs provided by the manufacturer—they are between 15–30 nm, as measured with the ImageJ software (Figure 1B).

Visible aggregates were formed for the MWCNT sample because MWCNT powder was dissolved in ethanol in order to apply them on the silica grid. MWCNT-Ox was already dissolved in PBS.

**Figure 2:**
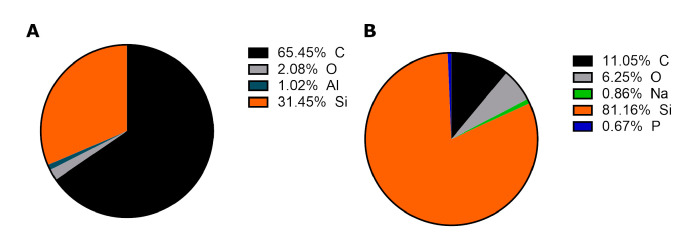
EDS analysis of MWCNTs (**A**) and functionalized MWCNT-Ox (**B**).

EDS analysis for MWCNTs (Figure 2A) showed aluminum impurities, which is the leftover after synthesis (most probably, an aluminum-based material was used as a support in CVD synthesis).

However, aluminum is not visible after functionalization (Figure 2B). The reaction reagents are concentrated strong acids—sulfuric and nitric, which dissolve the residual metal, and it is subsequently removed with the series of washes. The sodium and phosphorus come from the PBS—a buffer, in which the sample was dissolved. The signal from silicon is derived from the silicon mount used in SEM, and oxygen in the MWCNTs sample may derive from the ethanol used for solubilization of the MWCNTs in powder form to apply them on the mount.

**Figure 3:**
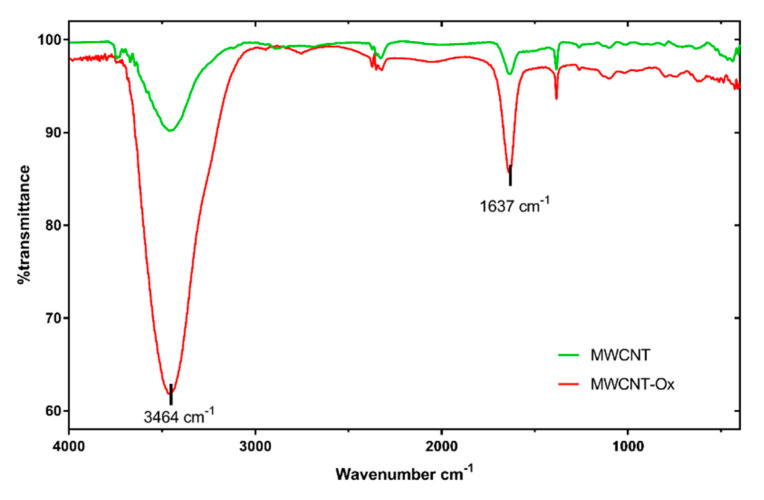
Fourier-transform infrared spectroscopy (FTIR) spectra of pristine MWCNTs and MWCNT-Ox.

FTIR spectra were obtained to compare surface groups on MWCNTs before and after functionalization (Figure 3).

There is a vast decrease in transmittance in two peaks in the MWCNT-Ox spectrum compared to pristine MWCNT. The first peak at 3464 cm^−1^ is a characteristic of the O-H stretch of a hydroxyl group, which can be ascribed to the oscillation of carboxyl groups. The peak at 1637 cm^−1^ corresponds to carbonyl groups, which were formed in the place of new defects and further oxidation during the functionalization reaction. They may also be a part of the carboxyl group. In conclusion, the FTIR spectra show that MWCNTs were oxidized.

### 3.2. Chondrocyte Viability

**Figure 4:**
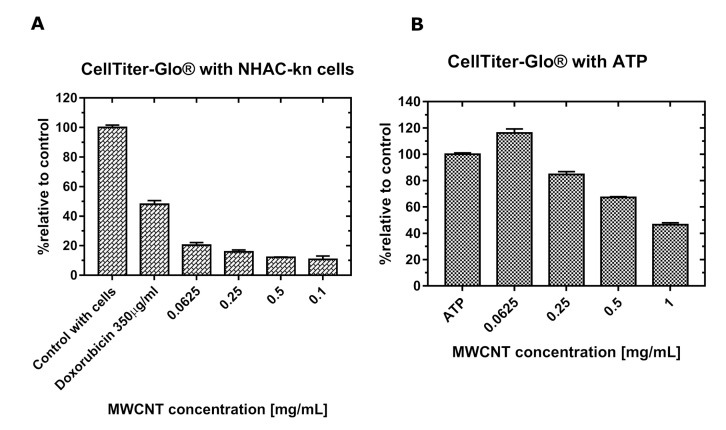
The plots represent relative cytotoxicity of MWCNT-Ox on NHAC-kn cells after 48 h (**A**) and signal of luminescence measured with the same concentrations of ATP and various concentrations of MWCNTs (**B**). Interference with the luminescent signal is visible. Doxorubicin as a positive control caused the death of over 50% of the cells.

After 48 h of incubation with MWCNT-Ox solutions, chondrocytes exhibited a significant decrease in the luminescent signal. At the lowest concentration, i.e., 0.0625 mg/mL, the measured signal shows that the viability of chondrocytes decreased by half compared to control, and it is decreasing with each subsequent concentration, suggesting the deaths of most of the cells (Figure 4).

### 3.3. ROS Assay

**Figure 5:**
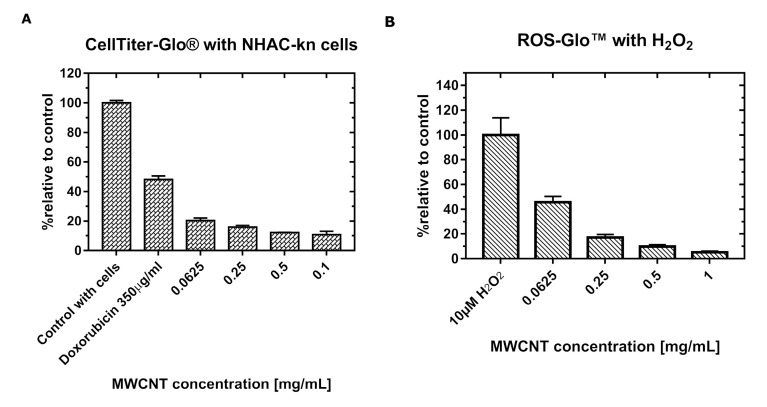
The plots represent the relative production of reactive oxygen species (ROS) in NHAC-kn cells after 48 h incubation with MWCNT-Ox (**A**) and signal of luminescence measured with the same concentrations of H_2_O_2_ and various concentrations of MWCNTs-Ox (**B**). A strong decrease in the luminescent signal is visible with increasing amounts of carbon nanotubes.

As in the test described above, the thermostable recombinant Ultra-Glo™ luciferase was used to convert the luciferin precursor to luciferin. As a positive control, 100 μM menadione—an organic chemical compound from the quinone group that induces the formation of reactive oxygen species was used, and this sample showed an over 4-fold increase in ROS production over control. The levels of hydrogen peroxide generation are shown in Figure 5.

After 48 h of incubation of the cells with the MWCNT-Ox solutions, in the lowest examined concentration (0.0625 mg/mL), the signal was higher than in control. Then, with increasing concentrations, the signal is strongly diminished, which may suggest very low H_2_O_2_ production. However, when this result is compared to the viability test (Figure 4), this may be attributed to the much lower viable cell number.

Moreover, the experiment with MWNCTs and the same concentrations of H_2_O_2_ revealed strong interference with the signal. There are two possible mechanisms of these interactions: (1) MWCNTs scatter a luminescent signal or (2) ROS are scavenged by the MWCNTs, and both of these actions can occur concomitantly. There is an indication that the latter mechanism occurs because the inhibition of the signal is stronger than measured with ATP.

### 3.4. Determination of Absorption and Luminescence Spectra

**Figure 6:**
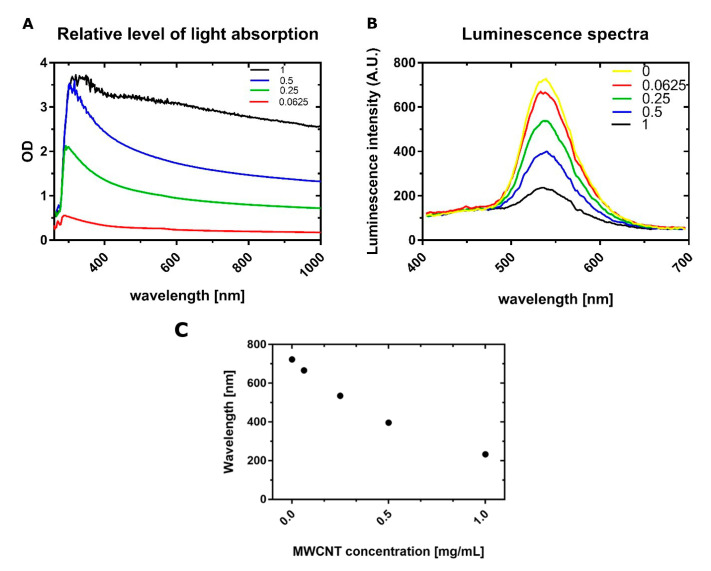
The Absorption spectrum of MWCNT-Ox used for experiments is shown on the (**A**) plot. The curves correspond to the respective concentrations of MWCNT-Ox. The maximum absorption is visible around 300 nm; however, it is observed throughout the whole range. The (**B**) plot shows luminescence spectra generated by the firefly luciferase from the CellTiter Glo assay, with a peak at λ = 532 nm. With an increasing amount of MWNCTs, the signal intensity is diminishing. When comparing both spectra, there is a visible overlap, which may indicate that some of the generated luminescent signals may be absorbed. The (**C**) plot represents a trend of decreasing maximum luminescence intensities with increasing concentrations of MWCNTs.

Recorded relative levels of absorbance show that relative absorbance increases significantly with increasing MWCNTs concentrations. As can be observed in Figure 6A, MWCNTs absorb a wide spectrum of light, which overlaps partly with the spectrum of light emitted by luciferase from CellTiterGlo assay (Figure 6B), with the maximum emission at λ = 532 nm.

Luminescence spectra were obtained for all of the concentrations used (0, 0.0625, 0.25, 0.5, and 1 mg/mL), and it can be seen that with the increasing amount of MWCNTs, there is a stronger decrease in signal intensity, which confirms that MWCNTs interfere with the luminescent signal.

### 3.5. Quenching of Luminescent Signal

For both tests, it is shown that MWCNTs quench the luminescent signal (Figure 4B and Figure 5B). The exact values of interference are shown in Table 1 and Table 2.

As the concentration of MWCNTs increases, the luminescent signal decreases compared to the control, despite the presence of the same amount of substance (1 μM ATP and 10 μM H_2_O_2_). One exception is in the 0.0625 mg/mL sample, where the luminescent signal is even higher than in the control.

These numbers could potentially be taken into account to correct the obtained results. However, using percentages alone would yield unreliable values, because they are calculated from the measured luminescence intensities, which were obtained with a fixed amount of analyzed substance. The simple addition or subtraction of intensities does not apply either, because they should be measured with the same ATP amount, as was present in the well after cell lysis. The only possible way to obtain reliable results would be a complete separation of released ATP from MWCNTs, which is not possible, due to a few reasons. Firstly, the supernatant with MWCNTs could be discarded before performing the assay; however, after doing so, some amount of MWCNTs are embedded in the matrix, which is secreted by the chondrocytes. The detachment of cells and centrifugation does not separate them from the nanotubes, because they are present in the pellet. Centrifugation after lysis would also lead to errors because some amount of ATP would be present in the pellet, either adsorbed on the surface of the nanotubes or with cellular debris.

## 4. Discussion

In this paper, the cytotoxicity of functionalized, multiwalled carbon nanotubes on NHAC-kn human chondrocytes was investigated by analyzing cell viability and the level of generated reactive oxygen species, using luminescence-based tests. This method of detection was chosen because in the previous studies, it was shown that carbon nanotubes interfere with absorbance and fluorescence-based assays. Tests utilizing luminescence as a measurement method offers a number of advantages, such as higher sensitivity due to lower background, the endogenous production of a signal, without the need of an external excitation source, and a broad wavelength spectrum of emitted light. At first glance, assays performed in this paper are showing that prolonged incubation with functionalized MWCNT-Ox significantly decreases the number of viable chondrocytes. Similar results were obtained when examining the effect of SWCNTs on the proliferation of kidney epithelial cells HEK293 [24], MWCNTs on the proliferation of skin epithelial cells [40], and A549 lung adenoma cells [41,42]. All of these studies indicate the toxicity of these nanomaterials. However, the experiment performed with the same concentrations of MWCNT-Ox and a fixed amount of a compound that is detected by the test (ATP) showed different signal intensities in each of the examined concentrations (Figure 4B). This result proves the interaction of MWCNTs with the assay. Exposure of cells to a solution of functionalized carbon nanotubes has shown to lead to the generation of ROS. Here, the ROS quantification assay was performed, and similar results as in the viability assay were observed (Figure 5A). With the lowest amount of MWCNTs, the assay reveals higher ROS generation than in control and a strong decrease with increasing concentrations. It was shown previously that carbon nanotubes may induce ROS production in cells; however, these values should be growing with higher carbon nanotube content. The decline shown here may be attributed to the much lower amount of viable cells. However, in an experiment, where only H_2_O_2_ was added to the MWCNT-Ox, an even stronger inhibition of signal was observed than in the analogous one with ATP. The possible explanation of this phenomenon is, additionally to luminescent signal absorption, the scavenging of generated ROS by the nanotubes. Additionally, absorption (Figure 6A) and luminescence spectra (Figure 6B) were obtained for all tested concentrations of MWCNT-Ox. MWCNTs showed a strong absorption of light, which partly overlaps with the luminescent spectrum. More importantly, the obtained luminescent spectra show a decrease in intensity with increasing MWCNT concentration. Therefore, the results of conducted experiments indicate that MWCNT-Ox interferes with the luminescent based-assays, yielding erroneous results.

To our knowledge, we are the first group to address the issue of the nanomaterials’ interference with the luminescent assays. Similar experiments were carried out by Pem et al., where the interference of lanthanide-doped nanoparticles with common in vitro toxicity assays based on absorbance and fluorescence was examined, including ROS generation assays [43]. Together with previously published discrepancies in absorbance and fluorescence-based assays [36,37], this research casts doubt on many of the already published papers. For example, Ursini et al. performed cytotoxic, genotoxic, and inflammatory response to carboxyl-modified MWCNT on A549 and BEAS-2B cells [44]. To evaluate toxicity, WST-1 assay was used. A severe decrease in viability after incubation with increasing MWCNT-COOH concentrations was observed, with the number of 28.5% of viable cells at the highest evaluated concentration. This is the same pattern as the data presented in this paper. In order to evaluate the reliability of these results, similar control experiments as in this paper should be carried out. In this case, a known amount of hydrophilic formazan crystal formed from WST-1 should be incubated with respective MWCNT-COOH concentrations to verify whether carbon nanotubes do not interfere with the signal, yielding a false-positive result. Recently, a similar experiment with WST-1 assay was carried out by Scarcello et al. on manganese nanoparticles, with the conclusion that luminescence-based tests could be more appropriate for this type of analysis [45]. This further confirms that research on the interference of nanomaterials with luminescence-based assays is needed.

Another important aspect of carbon nanotubes toxicity is the impurities, which are often remnants of metal catalysts used in the synthesis. Carbon nanotubes for this paper were provided by the company, claiming at least 95% purity; therefore, we performed EDS analysis (Figure 2) to check the possible content of residual impurities.

Indeed, provided MWCNTs contained some metal impurities; however, after the functionalization process, it was not detected on the EDS analysis. Hence, we do not account for any interference with our measurements from it.

Further toxicological studies on in vitro and in vivo models should be developed to learn more about the effects of CNTs on health and body function. It would be desirable to learn more about the mechanism of ROS generation at the level of gene expression. Understanding these mechanisms will allow a better understanding of the interaction of cells with nanotubes, and it may perhaps develop ways to functionalize nanotubes to minimize these negative effects. 

The interference of CNTs with the majority of assays is a serious issue. There is a growing number of research regarding 3D-cell scaffolds containing CNTs for application in regenerative medicine [46]. The viability of cells that are embedded in such structures is usually measured by these assays or their derivatives. In such composite materials, the CNTs are embedded in the scaffold. Therefore, they are non-removable before the assay, which may affect the final result, if CNTs are shown to interfere with the assay components. The lack of reliability of these tests casts even more emphasis on undertaking more in vivo studies, which should yield more trustworthy results. 

On the other hand, an animal experiment is preceded by dosage determination based on in vitro assays; therefore, this may lead to improper experiment design. What is more, there are few in vivo animal studies in the literature that can better illustrate the total response of the body upon exposure to nanotubes. Currently, these studies focus on pulmonary, intraperitoneal, and subcutaneous administration. The level of toxicity induced by MWCNTs on the respiratory system of rats was assessed by the endotracheal aspiration of solutions at various concentrations. The toxic effect was dependent on the dose of carbon nanotubes and increased over time [47]. Orally administered SWCNTs and MWCNTs did not cause death or toxic effects in the tested rats. It is suggested that carbon nanotubes in the digestive system have formed agglomerates that have been removed from the body in the undigested form [48]. In contrast, the local application of SWCNTs to the skin of mice caused inflammation at the application site and the occurrence of oxidative stress [49]. It would be also reasonable to administer CNTs intravenously or to specific organs or body parts. In conclusion, despite years of research carried out, owing to the complexity of carbon nanotubes interactions with biological matter, combined with their physical and chemical properties, the methodology in determining the mechanisms of toxicity still needs to be improved and shifted toward in vivo experiments.

## 5. Conclusions

The conducted research shows that oxidized MWCNTs interfere with the luminescent signal generated by a commercially available assay, CellTiter Glo. This was confirmed by a series of experiments, where both absorbance and luminescent spectra were obtained. There is an overlap in these spectra, which may suggest that a part of the luminescent signal is quenched by MWCNTs. Importantly, a series of luminescent spectra with various amounts of MWCNTs showed that there is a decrease in signal intensity with increasing MWCNTs concentrations. Further studies elucidating the exact mechanism of this interference may help to overcome the limitations of the currently used assay.

## Figures and Tables

**Table 1:** Table showing the amount of MWCNTs interference with the luminescent signal generated by the viability assay, with a fixed amount of ATP.

MWCNTs Concentration [mg/mL]	Percentage of Signal Relative to Control [%]	Amount of Interference [%]
0	100	-
0.0625	116.13	−16.13
0.25	84.63	+15.37
0.5	67.27	+32.73
1	46.51	+53.49

**Table 2:** Table showing the amount of MWCNTs interference with the luminescent signal generated by the ROS assay, with a fixed amount of H_2_O_2._

MWCNTs Concentration [mg/mL]	Percentage of Signal Relative to Control [%]	Amount of Interference [%]
0	100	-
0.0625	45.73	+54.27
0.25	17.03	+82.96
0.5	9.78	+90.21
1	5.19	+94.8
